# Motivational factors associated with intentions to pursue anesthesiology among medical students: a cross-national study

**DOI:** 10.3389/fmed.2026.1871796

**Published:** 2026-08-19

**Authors:** Marie Kratzer, Milena Abbiati, Nadia M. Bajwa, Georges L. Savoldelli

**Affiliations:** 1 Division of Anesthesiology, Department of Acute Care Medicine, Geneva University Hospitals, Geneva, Switzerland; 2 Department of Anesthesiology, Pharmacology, Intensive Care and Emergency Medicine, Faculty of Medicine, University of Geneva, Geneva, Switzerland; 3 Unit of Development and Research in Medical Education (UDREM), Faculty of Medicine, University of Geneva, Geneva, Switzerland; 4 Department of General Pediatrics at the Children’s Hospital, Geneva University Hospitals, Geneva, Switzerland

**Keywords:** anesthesiology, career choice, career intentions, medical students, motivational factors, specialty selection

## Abstract

**Introduction:**

Understanding what motivates students to choose a specialty can be a lever for shaping choices aligned with the needs of the healthcare system. This study aimed to determine students’ intentions to pursue anesthesiology and to identify the motivational factors associated with these intentions in two different training systems.

**Methods:**

This is a cross-sectional observational study (2018–2021) based on a self-administered questionnaire completed by final year medical students in France and Switzerland. Principal component analysis and Cronbach’s alpha were performed to provide preliminary evidence of internal structure and internal consistency of a revised version of the Motivational Career Choice Questionnaire (MCCQ-R). Logistic regression analysis was performed to identify demographic and motivational factors associated with anesthesiology career intentions.

**Results:**

A total of 703 participants were included (response rate 74, 62% women). The average proportion of undergraduates intending to choose anesthesiology was 7.7% (*n* = 54). French students were significantly less likely than Swiss students to report this intention [4.1% (*n* = 10) in Strasbourg vs. 10.3% (*n* = 23) in Lausanne and 8.8% (*n* = 21) in Geneva]. A particularly deterrent factor among French students was work-life balance (mean score 2.76 ± 1.8; *p* = 0.001). Principal component analysis identified three motivational factors labeled “prestige,” “diverse scope of practice” and “working conditions,” which explained 60% of the variance in motives. Logistic regression identified “diverse scope of practice” (OR 1.69 [1.25–2.29]; *p* = 0.001) and “working conditions” (OR 1.73 [1.22–2.45]; *p* = 0.002) as factors associated with anesthesiology career intentions.

**Conclusion:**

“Diverse scope of practice” and “working conditions,” as measured by the MCCQ-R, are associated with students’ intention to choose anesthesiology as a specialty. Emphasizing these aspects during medical training could help attract students to the specialty.

## Introduction

Aging populations and growing perioperative demands are increasing pressure on healthcare systems worldwide. Anesthesiology is among the specialties most affected by workforce shortages: a Swiss demographic survey projects that the number of newly certified anesthesiologists will be insufficient to replace retiring specialists (1), and comparable trends have been documented in France (2) and globally (3). While both countries have responded partly through foreign recruitment [over the past decade 74% of newly board-certified physicians in Switzerland trained abroad (4)] such reliance risks depleting resources in source countries already facing their own shortages (5). A more sustainable approach lies in better aligning medical students’ career choices with healthcare system needs, which requires understanding the motivational factors that shape specialty intentions (6).

Despite a broad literature on specialty choice, anesthesiology has received limited focused attention: it typically appears as one option among many in multi-specialty surveys (7), consistently attracts fewer students than surgery, internal medicine, or pediatrics (8–11). Studies specifically examining motivational determinants of anesthesiology career intentions remain scarce (12, 13), and students’ baseline knowledge of the anesthesiologist’s role has been shown to be limited prior to clinical exposure (14, 15). Structured analyzes of the motivational profiles associated with choosing anesthesiology, therefore, remain underrepresented in the literature (16, 17). Gender also influences specialty decision-making in medicine (6, 18). Although its specific role in the choice of anesthesiology remains underexplored, a recent Swiss survey documented a progressive feminization of the specialty in recent decades (1), suggesting that gender-related motivational patterns may be evolving and warrant closer examination.

An additional challenge is the limited availability of validated instruments to measure these factors. One notable exception is the Motivational Career Choice Questionnaire (MCCQ), originally developed to assess motives for choosing family medicine (19) and subsequently validated in French in a Canadian context (20). To our knowledge, it is the only such instrument validated in French.

Specialty choice may also be shaped by the regulatory framework governing postgraduate training. In Switzerland, graduates apply freely to the residency program of their choice. In France, specialty allocation once determined by a national ranking examination (ECN), was recently replaced by the National Dematerialized Examination (EDN), in which students are ranked across 13 specialty groups; pursuing anesthesiology requires strong performance in the acute medicine group. Both systems broadly respect the preferences of high-achieving students (21), and anesthesiology remains a competitive specialty in France (22). France thus represents a centralized, exam-based model common in several countries, while Switzerland illustrates a more flexible, application-based system found in other decentralized training contexts. To our knowledge, no study has directly compared motivational profiles for choosing anesthesiology across countries with contrasting regulatory frameworks.

This study addresses these gaps by examining anesthesiology career intentions of final-year medical students and identifying the motivational factors associated with these intentions in France and Switzerland, using a revised French version of the MCCQ adapted to anesthesiology (MCCQ-R). By comparing two countries with structurally different postgraduate training systems, this study may help inform strategies to attract future anesthesiologists, which is relevant to other high-income countries facing similar workforce challenges.

## Methods

### Study design and setting

This was a cross-sectional survey of final-year medical students (year 6) from two Swiss medical schools (Geneva and Lausanne) and from one French medical school (Strasbourg).

Students at all three universities were invited to complete a questionnaire at the end of year 6. Swiss medical students were surveyed in 2019 and 2021, whereas French students were surveyed in 2018. It should be noted that French students completed the questionnaire approximately 3 months before sitting the ECN. At the time of data collection, no French student had therefore yet been allocated to any specialty training pathway. This timing ensures that the motivational profiles capture pre-selection career intentions, comparable to those of the Swiss cohort. Before agreeing to participate in the study (by signing a consent form), students had been informed by email of the research project’s content, their rights and responsibilities as volunteer participants, and the terms of confidentiality and privacy.

### Questionnaire

The survey (electronic or paper questionnaire) was composed of three sections. The first section included sociodemographic data: age, gender, nationality, type of high school diploma, and two indirect measures of students’ socioeconomic level, i.e., parents’ highest educational achievement and parents’ profession.

The second section included students’ specialty career choice intentions [based on the list of the Swiss specialist titles issued by the Swiss Institute for Postgraduate Medical Training, plus an “undecided” category for Switzerland and the ECN specialist titles in France (23, 24)], as well as students’ level of motivation for their specialty choice using a six-point Likert scale (1 = very unmotivated, 6 = highly motivated).

In the third section, we used the MCCQ-R, a revised version of Wright’s Motivational Career Choice Questionnaire (19) that had been validated in French by Beaulieu (20). The original English MCCQ included 25 items, and its factorial structure had been confirmed in previous studies. The French version, developed in Quebec, retained 17 items from the original and added three new items related to future practice setting and residency duration (20); its factorial structure was also confirmed. For the present study, we further revised the French version, resulting in the MCCQ-R, which includes 12 items rated on a six-point Likert scale (1 = “very dissuasive” to 6 = “very attractive”). Among these, seven items were retained from the previous French version: “Working in a rural setting/outlying hospital,” “Shortage in the specialty,” “Type of patient follow-up (long-term relationship),” “Potential income,” “Duration of postgraduate training,” “Social acknowledgment,” and “Peer acknowledgment.” The remaining five items were newly combined or adapted for relevance to anesthesiology: “Lifestyle/family-work balance” (combining two original items on work hours), “Variety of pathologies” (combining two items on diversity of pathologies), “Intellectual challenge/level of complexity,” “Career development opportunities,” and “Role model.” To establish content validity, a multidisciplinary expert panel comprised of a PhD psychologist, a PhD educational scientist, and three acute care physicians supervised the shortening and adaptation of the original MCCQ into the 12-item revised version (MCCQ-R). This panel ensured that the selected items captured all relevant motivational dimensions of the field, while eliminating redundant items from the original primary care questionnaire. This adapted version was previously pilot-tested among Master’s medical students across the three participating sites, demonstrating satisfactory readability and preliminary psychometric stability (25). Given the exploratory nature of this study, these analyzes provide preliminary evidence of internal structure and internal consistency within this sample, rather than a definitive validation of the instrument.

### Ethical considerations

This study was exempted in Switzerland from a full ethical review by the Chair of the Cantonal Commission for Ethical Research (CCER—protocol 2020-00813) and by the Institutional Review Board of Strasbourg medical school (#2020-2017-25). The purpose of the study was presented to all medical students via email and orally on the day of the survey administration. Participation was voluntary, and all survey responses were anonymous.

### Statistical analysis

We used descriptive statistics for demographics, specialty choice, and students’ perceptions of the attractiveness of anesthesiology. Chi^2^ and ANOVA were used for comparisons between universities, and the type I error rate was set at 0.05. The normality of continuous variables was assessed using skewness, kurtosis, and visual inspection of Q-Q plots; given the large sample size (*N* = 703), parametric analysis was deemed appropriate as all distributions were reasonably symmetric. Homogeneity of variances was tested using Levene’s test, and Welch’s ANOVA correction was applied to the few variables where this assumption was violated.

We used principal component analysis (PCA) with Varimax rotation to aggregate the 12 motives with a frequency of >10%. Data suitability was confirmed using the Kaiser-Meyer-Olkin (KMO) sampling adequacy index. Combined criteria (scree plot, eigenvalue >1.0, and interpretability) were used to determine the number of factors. The critical value for significant factor loading was set at >0.40 (26). We labeled each factor obtained from the PCA procedure to reflect its content, following previous MCCQ versions (19, 20), and we also calculated Cronbach’s alpha for each factor to assess internal consistency. This analysis was performed to confirm the factorial structure in this specific population, as this tool was slightly revised and applied for the first time to anesthesiology.

We used logistic regression analyzes (Odds Ratios and 95% confidence intervals) to determine whether gender, country, and motivational factors obtained from the PCA were associated with the intention to choose anesthesiology as a career (0 = no intention; 1 = intention to choose anesthesiology). To avoid model overfitting and preserve statistical power, a parsimonious set of five predictors (gender, country, and the three extracted motivational factors) was selected based on *a priori* relevance in the literature (27). Specifically, previous literature on medical specialty choice suggests that intentions toward anesthesiology are often influenced by domains such as working conditions and lifestyle (15), the clinical scope of practice and intellectual challenge (28), and professional prestige or recognition (14). These domains, described in prior studies, align with the three primary factors extracted from our PCA, supporting their selection as predictors in the multivariable model. Potential confounding factors such as age and nationality were not included because of the high homogeneity of cohort. Clinical exposure was omitted to prevent severe multicollinearity with the country variable, as it is structurally nested within each national curriculum. In the final model, the ‘country’ variable effectively controls for these baseline systemic differences, ranging from mandatory combined rotations in Anesthesiology and Intensive Care in France to elective-based exposure in Switzerland. The final model yielded an Events Per Variable (EPV) ratio of approximately 11:1, satisfying standard requirements for stability. Multicollinearity was formally assessed using the Variance Inflation Factor (VIF), with all values remaining well below 2.5, and the model’s goodness of fit was confirmed to be satisfactory according to the Hosmer–Lemeshow test, *χ*^2^(8) = 6.59, *p* = 0.582. Statistical analyzes were conducted with SPSS version 24 (IBM Corp., Armonk, NY, USA).

## Results

### Participants

Of 938 final year students, 703 fully completed the questionnaire. The overall response rate was 74.9% (*n* = 703/938): 77.0% (*n* = 238/309) in Geneva, 58.8% (*n* = 223/379) in Lausanne, and 96.8% (*n* = 242/250) in Strasbourg. The distribution of participants across study sites was 33.8% (*n* = 238) in Geneva, 31.7% (*n* = 223) in Lausanne, and 34.4% (*n* = 242) in Strasbourg. The overall proportion of women was 62.4% (*n* = 439) with no difference found between the three universities (*n* = 147 in Geneva, *n* = 138 in Lausanne and *n* = 154 in Strasbourg, *p* = 0.689). There were no differences regarding students’ age between universities (overall mean = 26, *p* = 0.247).

### Specialty choice

Table 1 shows the distribution of students’ intentions to choose anesthesiology by university and country, as well as the gender distribution. The overall intention of choosing anesthesiology was 7.7%. These rates ranged from 10.3% in Lausanne, 8.8% in Geneva versus 4.1% in Strasbourg (*p* = 0.001). Gender distribution was similar in both countries and across all medical schools.

**Table 1:** Students’ intentions and level of motivation to choose anesthesiology by faculty.

Variable	Total	Swiss faculties		French faculty	*p*-value
	*n* = 703	Geneva *n* = 238	Lausanne *n* = 223	Strasbourg *n* = 242	
Intention to choose anesthesiology	54 (7.7)	21^a^ (8.8)	23^a^ (10.3)	10^b^ (4.1)	0.020
Intention to choose anesthesiology by gender (Reference: Male)	27 (50)	12^a^ (57.1)	10^a^ (43.5)	5^a^ (50)	0.216
Motivation for anesthesiology	2.7 ± 1.6	2.9^a^ ± 1.7	2.9^a^ ± 1.6	2.4^b^ ± 1.6	0.001

Variables are expressed as numbers (percentages) and means ± standard deviations.

Each index letter specifies a subset of categories which proportions/means per column do not differ significantly at a *p* level of 0.05.

Table 1 also shows students’ average level of motivation to become an anesthesiologist. French students had the lowest score (2.4 ± 1.6 versus 2.9 ± 1.7 in Geneva and 2.9 ± 1.6 in Lausanne, *p* = 0.001).

To address a potential confounding effect of the COVID-19 pandemic, an internal chi-square analysis comparing the Swiss cohorts surveyed before (2019) and during (2021) the pandemic revealed no statistically significant difference in intentions (*p* = 0.231). Furthermore, when questioned directly, only a minor proportion of students across both countries reported that COVID-19 had influenced their career choice.

### Motives to choose anesthesiology

Table 2 shows the mean ± Standard Deviation (SD) of the twelve MCCQ-R motives by faculty. For all subsequent comparisons between French and Swiss students, the observed differences were highly significant (all *p* = 0.001). French students found the following motives more attractive than Swiss students did: “variety of pathologies,” “intellectual challenge/level of complexity,” “career development opportunities,” “social acknowledgment,” and “peer acknowledgment.” Conversely, the motives “lifestyle/family-work balance” and “working in a rural setting/outlying hospital” were more dissuasive for French students. Between the two Swiss faculties, only two significant differences were found (both *p* = 0.001): “career development opportunities” were more attractive for students from Geneva, whereas “working in a rural setting/outlying hospital” was more dissuasive to them. However, overall, none of these differences exceeded ±1 standard deviation, except for the ‘lifestyle/family-work balance’ motive.

**Table 2:** Motives influencing intention to choose anesthesiology by faculty.

Motives	Swiss faculties		French faculty	Overall (all faculties)	*p*-value
	Geneva	Lausanne	Strasbourg		
Intellectual challenge/level of complexity	4.49^a^ ± 1.3	4.31^a^ ± 1.4	4.94^b^ ± 1.3	4.59 ± 1.4	0.001
Potential income	4.38^a^ ± 1.1	4.27^a^ ± 1.2	4.51^a^ ± 1.2	4.39 ± 1.2	0.090
Career development opportunities	4.09^a^ ± 1.3	3.84^b^ ± 1.4	4.32^c^ ± 1.2	4.09 ± 1.3	0.001
Lifestyle/family-work balance	4.02^a^ ± 1.6	4.02^a^ ± 1.6	2.76^b^ ± 1.8	3.58 ± 1.8	0.001
Peer acknowledgment	3.76^a^ ± 1.2	3.54^a^ ± 1.3	4.61^b^ ± 1.3	3.98 ± 1.3	0.001
Social acknowledgment	3.70^a^ ± 1.2	3.50^a^ ± 1.2	4.57^b^ ± 1.2	3.94 ± 1.3	0.001
Duration of post-graduate training	3.53^a^ ± 1.2	3.41^a^ ± 1.1	3.38^a^ ± 1.2	3.44 ± 1.2	0.338
Variety of pathologies	3.50^a^ ± 1.6	3.23^a^ ± 1.5	4.45^b^ ± 1.5	3.74 ± 1.6	0.001
Working in a rural setting/outlying hospital	3.29^a^ ± 1.4	3.62^b^ ± 1.4	2.95^c^ ± 1.4	3.27 ± 1.4	0.001
Role model	3.16^a^ ± 1.6	2.83^a^ ± 1.3	3.01^a^ ± 1.4	3.00 ± 1.5	0.052
Type of patient follow-up (long-term relationship)	3.09^a^ ± 1.6	2.87^a^ ± 1.5	2.82^a^ ± 1.5	2.93 ± 1.6	0.115
Shortage in the specialty	2.92^a^ ± 1.2	3.04^a^ ± 1.2	3.11^a^ ± 1.3	3.03 ± 1.2	0.267

Variables are expressed as means ± standard deviations.

Each index letter specifies a subset of categories which proportions/means per column do not differ significantly at a *p* level of 0.05.

### Principal component analysis

Using PCA, the aggregation of the 12 motives included in the MCCQ-R revealed that three principal components (or factors) accounted for 60% of the variance related to the motives for choosing anesthesiology as a career (Table 3). Factor 1 was labeled “prestige,” as it included “peer acknowledgment,” “social acknowledgment,” “potential income,” and “role model,” and represented 23.2% of the variance (Cronbach’s alpha = 0.76). Factor 2 was renamed “diverse scope of practice”; it regrouped “variety of pathologies,” “intellectual challenge/level of complexity,” “type of patient follow-up,” and “career development opportunities,” and explained 20.6% of the variance (Cronbach’s alpha = 0.79). This latter motive also co-loads on Factor 1, but is weaker than on Factor 2. Finally, Factor 3 was renamed “working conditions” since it combined “lifestyle/family-work balance,” “working in a rural setting/outlying hospital,” “duration of postgraduate training,” and “shortage in the specialty” and accounted for 16.81% of the variance (Cronbach’s alpha = 0.69).

**Table 3:** Principal component analysis (varimax with Kaiser normalization) aggregating the twelve Motivational Career Choice Questionnaire Revised (MCCQ-R) motives.

Motives	Component loading	Motivational factors (% variance; eigenvalue)
Peer acknowledgment	0.89	1. Prestige (23.25; 2.8)
Social acknowledgment	0.88	
Potential income	0.60	
Role model	0.41	
Variety of pathologies	0.86	2. Diverse scope of practice (20.64; 2.5)
Intellectual challenge/level of complexity	0.73	
Type of patient follow-up (long-term relationship)	0.72	
Career development opportunities	0.56	
Lifestyle/family-work balance	0.90	3. Working conditions (16.81; 2.0)
Working in a rural setting/outlying hospital	0.59	
Duration of postgraduate training	0.51	
Shortage in the specialty	0.49	

### Association with the intention to choose anesthesiology

As highlighted in Table 4, the logistic regression analysis showed that two motivational factors were significantly associated with the intention to choose anesthesiology as a specialty: the factor “diverse scope of practice” (OR 1.69; 95% CI [1.25–2.29]) and the factor “working conditions” (OR 1.73; 95% CI [1.22–2.45]). The variables “gender,” “country of study,” and the factor “prestige” were not correlated with this intention. Multicollinearity was formally assessed using the Variance Inflation Factor (VIF), with all values remaining well below 2.5. Model fit was satisfactory, as indicated by a non-significant Hosmer–Lemeshow test, *χ*^2^(8) = 6.59, *p* = 0.582.

**Table 4:** Factors independently associated with “intention to choose anesthesiology” after multivariate logistic regression.

Variable	OR [95% CI]	*p*-value
Gender (*reference: male*)	0.65 [0.37–1.17]	0.151
Country (*reference: France*)	1.72 [0.77–3.80]	0.184
Prestige	0.86 [0.62–1.18]	0.343
Diverse scope of practice	1.69 [1.25–2.29]	0.001^*^
Working conditions	1.73 [1.22–2.45]	0.002^*^

OR, Odds ratio; CI, Confidence interval.

Binary variables were coded as follows: Gender (0 = male [reference], 1 = female) and country (0 = France [reference], 1 = Switzerland).

^*^A *p*-value of <0.05 was considered significant.

## Discussion

The overall proportion of students intending to choose anesthesiology was 7.7%. Internationally, the proportion of medical students intending to pursue anesthesiology varies considerably across educational contexts. Available data report rates of approximately 8% as a first-choice specialty among final-year students in South Africa (29), 8% pre-clerkship in Pakistan, and up to 24% following clinical exposure (30), and approximately 8.3% across six countries in a recent systematic review (13). Our results were within the lower side of the reported range. They also mirrored the proportions of a previous nationwide Swiss study covering 2011–2014, confirming that there was not a substantial increase in the number of students intending to specialize in the field (31). Our results also revealed a difference in intention rates between France (4.1%) and Switzerland (9.6%). This may be partly due to the differences in specialty regulation systems. Swiss residents can still “easily” change their minds during their postgraduate training and reorient their career choices. In France, on the other hand, while the procedure is possible (“droit au remords”), it is much more closely supervised and regulated. Interestingly, despite these two different regulatory systems, the proportion of anesthesiologists in the workforce is similar. In 2021, the anesthesiology workforce represented 4.5% of the total number of physicians practicing in Switzerland (32), resulting in 18.3 anesthesiologists per 100,000 inhabitants (33). The same year, in France, anesthesiologists represented 5.2% of practicing physicians, or 12.4 anesthesiologists per 100,000 inhabitants (34).

Consequently, considerations relating to the imbalance in the anesthesiology workforce are similar in both countries, mainly due to the aging population (35). Concerns about the shortage of anesthesiologists became more tangible during the recent pandemic, due to the role anesthesiologists played in intensive care units and the dramatic increase in the need for such specialists (36). The anesthesiology workforce shortage post-pandemic was confirmed worldwide (37). The present study does not directly test workforce adequacy or longitudinal career outcomes. However, the low proportion of final-year students in this sample who intend to pursue anesthesiology, provides contextually relevant observational data that may complement existing demographic projections. Whether this proportion will ultimately translate into definitive career choice depends on factors beyond the scope of this study, including residency availability, match outcomes, and specialty attrition rates during training.

Our results showed that the current lifestyle and work-family balance offered by anesthesiology seem to discourage career intentions for this specialty in France. This confirms an earlier study, which also showed that this motive is an increasingly strong deterrent to the choice of acute specialties during the pregraduate years (25). In contrast, our results did not support the influence of “role models” on student career choices in both countries. This contrasts with previous studies (38) and theories in the literature (6) on the importance of role models in career decision-making. We suggest that more research is needed to better understand its real impact.

A previous study reported that the motives “intellectual challenge/level of complexity” as well as “career development opportunities” were influential in the intention to choose anesthesiology (28). In our study, these two motives were among the most attractive for students and were higher for French than Swiss students. “Working in a rural setting/outlying hospital” appeared to deter French students compared to Swiss students. This could be explained by geographical differences between the two countries. Population density is higher in Switzerland, and most rural areas are not that far from urban areas compared to some French regions.

The perception of the specialty may also evolve depending on the stage of the training program, for example between medical students and residents. This is illustrated by a recent French study of junior anesthesiology residents, which found that social and professional recognition were considered the least important aspects of anesthesiologists’ professional lives (22). This is in contrast with our results showing that “variety of pathologies,” “social acknowledgment” and “peer acknowledgment” are three motives that make anesthesiology more attractive for French students.

To our knowledge, our study is the first to use the MCCQ to examine final-year medical students’ intentions to pursue anesthesiology. Our findings provide preliminary evidence of internal structure and internal consistency for this revised questionnaire. Notably, the PCA identified three motivational factors explaining a higher proportion of the variance than in the previous Wright and Beaulieu’s models (60% versus approximately 50%). These results offer a solid psychometric foundation within our sample, paving the way for a broader validation. To this end, we are currently expanding studies initiated a decade ago to comprehensively assess the measurement properties of this revised version for anesthesiology and other specialties across all Swiss medical faculties (25).

Our study identified two motivational factors that correlate with the intention to choose anesthesiology in both countries: “diverse scope of practice” and “working conditions.” Concerning the “diverse scope of practice,” this finding is in accordance with previous studies (1, 39). This factor gathers intellectual challenge, clinical variety, and career development opportunities, therefore from an educational perspective, increasing early and meaningful exposure to perioperative medicine, simulation-based learning, and multidisciplinary collaboration may reinforce these perceived strengths. “Working conditions” in anesthesiology are quite unique compared to other specialties. Clinical practice is always linked to a healthcare center, long-term patient follow-up is rare, and the administrative workload is lower than in other specialties. As reported in a survey conducted in England, many students today consider work-life balance important in their decision-making, and this aspect is strongly related to the workplace environment (40). Transparent communication regarding scheduling models, team-based practice, and hospital-based workflow may therefore contribute to specialty attractiveness.

“Prestige” is a factor that generally influences the choice of surgical specialties (31); this factor was not correlated with the intention to choose anesthesiology in our study. This is in line with previous studies concerning the choice of general medicine (19, 20). This could be the subject of future studies to better situate anesthesiology among other specialties.

Our results revealed no influence of gender on students’ intention to choose anesthesiology. This is in contrast with other specialties, such as surgery (41), pediatrics (18) or gynecology (31), for which gender was found to play an important role. Despite the overall underrepresentation of women in medicine worldwide, this disparity is no longer observed in medical schools (42). The feminization of anesthesiology has been reported (43). In France, this tendency is described as moderate compared with other specialties, such as pediatrics or gynecology (35); in Switzerland, the feminization of anesthesiology is clearly reported (44). Overall, our results suggest that gender balance in anesthesiology is well on the way to being achieved.

The present study has several limitations. Firstly, our study was limited to three French-speaking institutions (one in Strasbourg, France and two in both Geneva and Lausanne, Switzerland). The Strasbourg Faculty of Medicine’s ECN 2018 specialty preference distribution is broadly consistent with national aggregate data (45). In Switzerland, Geneva and Lausanne represent the majority of French-speaking Swiss medical graduates, however, German- and Italian-speaking Swiss faculties are not represented. The findings should therefore be interpreted as pertaining to French speaking European academic medical institutions with comparable undergraduate training structures. We encourage replication in more diverse institutional samples before broader national or international conclusions are drawn. While our response rate is very good, self-selection bias cannot be formally excluded, as is the case for any voluntary survey-based designs and should be considered when interpreting the findings. Additionally, due to sample size constraints regarding the number of participants, we could not account for potentially relevant socioeconomic background factors (SES). Future large-scale cohort studies should investigate whether socioeconomic status further influences undergraduate intentions toward anesthesiology. Regarding the temporal mismatch and the potential confounding effect of the COVID-19 pandemic, our data suggest that the health crisis did not significantly bias students’ career intentions toward anesthesiology. This is consistent with previous research within these specific educational contexts indicating that while the pandemic increased student anxiety, particularly in the French cohort, it did not fundamentally reshape specialty preferences (46). Lastly, our study design was a cross-sectional survey focused on intention rather than on definitive career choice limiting the inferences that we can make from our results. While the logistic regression identifies statistically significant associations between motivational factors and anesthesiology career intentions; no causal relationship can be inferred. Future large-scale prospective studies that track students from undergraduate training through residency application and specialty would be useful to better understand how motivational profiles evolve over time and whether they predict definitive career outcomes. The questionnaire was nevertheless completed at the very end of the final year, which in France was 3 months before the final assignment. Moreover, as a cross-cultural study, we believe this information may be useful to countries or regions struggling to attract an adequate workforce into anesthesiology.

## Conclusion

The students’ intentions to choose anesthesiology were within previously reported international ranges. The current proportion is unlikely to meet the future healthcare system’s or the population’s increasing needs for new professionals. The two residency regulation systems do not appear to have much influence on career motivational factors for choosing anesthesiology. The MCCQ-R yielded reliable scores for these motivations and could be used to assess the impact of future interventions. Emphasizing the diverse scope and working conditions of anesthesiology during medical school could help attract students to this specialty.

## Data Availability

The raw data supporting the conclusions of this article will be made available by the authors, without undue reservation.
